# Spatiotemporal Dynamics of Carbon Footprint of Main Crop Production in China

**DOI:** 10.3390/ijerph192113896

**Published:** 2022-10-26

**Authors:** Jianling Fan, Dengwei Guo, Lu Han, Cuiying Liu, Chuanhong Zhang, Jianan Xie, Junzhao Niu, Liwen Yin

**Affiliations:** 1 Jiangsu Key Laboratory of Atmospheric Environment Monitoring and Pollution Control, Jiangsu Collaborative Innovation Center of Atmospheric Environment and Equipment Technology, School of Environmental Science and Engineering, Nanjing University of Information Science and Technology, Nanjing 210044, China; 2 Jiangsu Key Laboratory of Agricultural Meteorology, School of Applied Meteorology, Nanjing University of Information Science and Technology, Nanjing 210044, China; 3 Reading Academy, Nanjing University of Information Science and Technology, 219 Ningliu Road, Nanjing 210044, China

**Keywords:** agricultural carbon footprint, life cycle assessment, greenhouse gas emission, crop production, mitigation

## Abstract

As a major agricultural country, the comprehensive accounting of the dynamics and composition of the carbon footprint of major crops in China will provide a decision-making basis for environmental management and agricultural green development in the whole process of the major crop production system in China. To investigate the spatiotemporal dynamics of the carbon footprint for major crops in China, a life cycle-based carbon footprint approach was used to evaluate the carbon footprint per unit area (*CF_A_*) and per unit yield (*CF_Y_*) of eight crops for the period of 1990 to 2019. Our results showed that the *CF_A_* for all major crops showed an increasing trend with time before 2016 but slowly decreased afterward, while the *CF_Y_* decreased by 16–43% over the past 30 years due to the increase in crop yield. The three main grain crops, rice (4871 ± 418 kg CO_2_-eq · ha^−1^), wheat (2766 ± 552 kg CO_2_-eq · ha^−1^), and maize (2439 ± 530 kg CO_2_-eq · ha^−1^), showed the highest carbon footprint and contribution to the total greenhouse gas (GHG) emissions, mainly due to their larger cultivated areas and higher fertilizer application rates. CH_4_ emission was the major component of the carbon footprint for rice production, accounting for 66% and 48% of the *CF_A_* and *CF_Y_*, respectively, while fertilizer production and usage were the largest components of carbon footprint for dryland crops, making up to 26–49% of the *CF_A_* and 26–50% of the *CF_Y_* for different crops. The present study also highlighted the spatial and temporal patterns of the carbon footprint for major crops in China, which could serve as references for the development of best management practices for different crop production in China, to mitigate agricultural GHG emission and to pursue low-carbon agriculture.

## 1. Introduction

Agriculture generates around a quarter of the global greenhouse gas (GHG) emissions [1], including 52% and 84% of global anthropogenic CH_4_ and N_2_O emissions, respectively [2]. The agricultural sector, however, is vital to human beings by providing a large number of products and services. With 18% of the global population, China holds the world’s largest agriculture market, with a 46.3% increase in crop yields from 2000 to 2015 [3,4]. Agricultural development in China has involved a high input of chemical fertilizers, pesticides, and agricultural film in the process of pursuing the increase in productivity, which lead to serious negative environmental effects, including the damage to natural resources, decrease in land productivity, accelerated spread of pests and diseases, and reduction in biodiversity [5,6]. Therefore, it is of great importance to analyze GHG emissions from crop production to make climate-resilient strategies for agricultural development in China.

Life cycle assessment (LCA) is a methodology for assessing the environmental aspects and potential impacts associated with a product [7]. Agricultural LCA comprehensively summarizes the impact of agriculture on the environment, which is an effective tool to promote the sustainability and green development of agriculture. In recent years, agricultural LCA has been widely used in agroecosystems for resource and environmental impact analysis, such as in intensive agriculture systems [8,9,10] and in organic agriculture [11,12,13]. The impact categories of the environmental load covered in LCA include climate change, ozone depletion, acidification, freshwater eutrophication, marine eutrophication, human toxicity, photochemical oxidant formation, particulate matter formation, terrestrial ecotoxicity, freshwater ecotoxicity, marine ecotoxicity, ionizing radiation, urban land use, and fossil energy consumption [14].

Recently, carbon footprint has been widely adopted as an efficient environmental indicator for climate change feedbacks, which is defined as the direct and indirect CO_2_ emission of a product or service in the life cycle process [15]. The carbon footprint calculation includes all GHG emissions as CO_2_ equivalents (CO_2_-eq), which permits the quantification of the whole life cycle of a product manufactured within a certain system [16]. Cheng, et al. [17] used national statistical data to evaluate the carbon footprint of crop production in China from 1993 to 2007, and found that the carbon emission of crop production in China was up to 119.5 Mt in 14 years, and the carbon footprint per unit area (*CF_A_*) was 0.78 ± 0.08 t CO_2_-eq ha^−1^. Yan, et al. [18] calculated the carbon footprint of major grain crops in China by a questionnaire survey of farmers in the representative region of eastern China. The *CF_A_* of rice, wheat, and maize were 6.0 ± 0.1, 3.0 ± 0.2, and 2.3 ± 0.1 t CO_2_-eq ha^−1^, respectively, while the carbon footprint per unit yield (*CF_Y_*) were 0.8 ± 0.02, 0.66 ± 0.03, and 0.33 ± 0.02 kg CO_2_-eq kg^−1^, respectively. Hillier, et al. [19] reported the carbon footprint for major crops in the UK and found that over 75% of the carbon footprint resulted from fertilizer application. Heidari*,* et al. [20] estimated the carbon footprint of durum wheat production in Iran and found that the variation in crop yield accounted for the majority of the variation in the carbon footprint. However, there are limited studies on the long-term spatial dynamic change of the agricultural carbon footprint and its composition assessment.

To quantify the mitigation potential of management strategies in China’s agriculture, we evaluated the spatiotemporal dynamics of the carbon footprint for major crops in China from 1990 to 2019, based on national and local statistical data. In order to provide a decision-making basis for the whole process of environmental management of China’s main crop production system, the influencing factors on the carbon footprint for major crop production were further analyzed. The specific objectives were: (1) to quantify the temporal and spatial variation in the carbon footprint of major crop production in China over the last 30 years and (2) to identify the main influencing factors on carbon footprint for different crop production systems in different regions.

## 2. Materials and Methods

### 2.1. The Study Area and Main Crops in Different Provinces

The carbon footprints of major crop production were calculated for 31 provinces of China from 1990 to 2019. Over the past 30 years, the averaged total sown area of crops in China has been 156,800 ha, among which eight major crops, including rice, wheat, maize, soybean, rapeseed, peanut, cotton, and highland barley, accounted for 62% to 96% of the total sown area for different provinces (Table S1). Therefore, we took the main crops from each province (Table S1) as the research objects and calculated the spatiotemporal dynamics of their carbon footprint.

### 2.2. Inventory Data Collection

Agricultural input data of major crops mainly include chemical fertilizers, seeds, pesticides, fuel, agricultural machinery and tools, and irrigation electricity consumption. Crop yields were calculated from the sown area and total yield from the China Statistical Yearbook series throughout the period of 1990–2019 [21]. The input data of chemical fertilizer and seeds for each province were available from the National Agricultural Product Cost and Income Data Compilation [22]. Pesticides, fuels, and irrigation power consumption were calculated indirectly through the National Agricultural Product Cost and Income Data Compilation [22] and the Price Yearbook of China [23]. Agricultural machinery and related parameters were obtained from the agricultural machinery network (https://toutiao.nongjitong.com/ (accessed on 20 April 2022). The emission factors of agricultural inputs mainly came from Ecoinvent database [24] and related references [25] (Table S2).

### 2.3. System Boundaries and Functional Units

**Figure 1:**
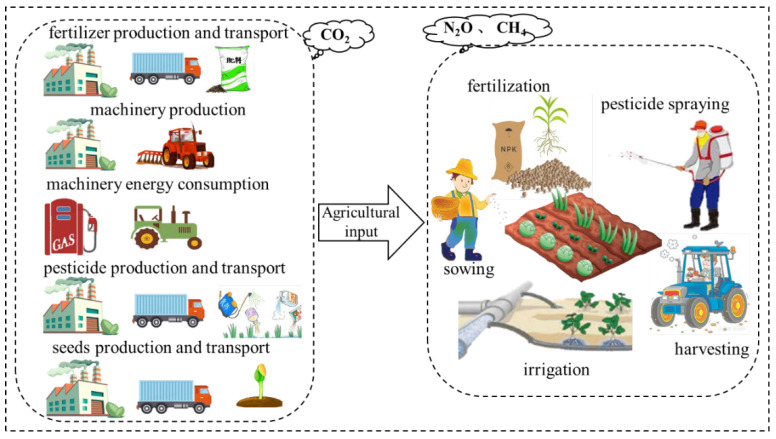
The system boundary of major crop production in China.

Total GHG emissions throughout the whole crop production process were quantitatively assessed in this study. The system boundary of crop production in this study was defined as the whole life cycle from the process of mining, production, and transportation of agricultural materials (chemical fertilizers, seeds, pesticides, etc.), to the completion of crop harvesting (Figure 1). Therefore, the sources of GHG emissions considered in this study, included the production, storage, transportation, and application of agricultural inputs, the energy consumption for machinery operation, CH_4_ emission from paddy fields, and N_2_O emission from N fertilizer application. The functional units (FU) were defined based on both the area and product in this study, i.e., the kg CO_2_-eq ha^−1^ of sown area and kg CO_2_-eq kg^−1^ of crop yield. FU based on area were used to compare the environmental effects, while FU based on the product were used to compare the production efficiency.

### 2.4. Carbon Footprint Calculation

Total GHG emissions from agricultural inputs in the carbon equivalent (*CE_input j_*, kg CO_2_-eq · ha^−1^) for crop *j* were estimated using the equation:

(1) $$CE_{input,j}=\sum_{i=1}^{n}\left(D_{i,j}\times EF_{i,j}\right)$$

where *D_i,j_* is the input of the *i*th agricultural material for crop *j* (kg ha^−1^ or kWh ha^−1^) and *EF_i,j_* is the emission factor of the *i*th agricultural material for crop *j* (kg kg^−1^ or kg GJ^−1^).

In particular, total N_2_O emission, including direct and indirect emissions, from agricultural land in the carbon equivalent for crop *j* (*CE_N2O,j_*, kg CO_2_-eq · ha^−1^) were calculated using the equation:

(2) $$CE_{N_{2}O,j}=\left(F_{D,N_{2}O,j}+F_{A,N_{2}O,j}+F_{L,N_{2}O,j}\right)\times 265$$

where *F_D,N2O,j_* (kg N_2_O ha^−1^) is the direct N_2_O emission from chemical N fertilizer application for crop *j*; *F_A,N2O,j_* and *F_L,N2O,j_* (kg N_2_O ha^−1^) are the indirect N_2_O emission caused by atmospheric deposition, leaching, and runoff, respectively, for crop *j*; and 265 is the global warming potential value of N_2_O at the 100-year time horizon [26].

Direct N_2_O emission *F_D,N2O,j_* for crop *j* (kg N_2_O ha^−1^) was calculated using the equation:

(3) $$F_{D,N_{2}O,j}=N_{input,j}\times F_{1,j}\times 44/28$$

where *N_input,j_* (kg ha^−1^) is the amount of N fertilizer application for crop *j* in farmland; *F*_1*,j*_ (kg N_2_O-N kg^−1^) is the N_2_O direct emission factor caused by N fertilizer application for crop *j*; and 44/28 is the ratio of N_2_O-N to N_2_O molecular weight.

Indirect N_2_O emission *F_A,N2O,j_* and *F_L,N2O,j_* (kg N_2_O ha^−1^) for crop *j* were calculated using the equations:

(4) $$F_{A,N_{2}O,j}=N_{input,j\;}\times R_{A,j}\times F_{2,j}\times 44/28$$

(5) $$F_{L,N2O,j}=N_{input,j}\times R_{L,j}\times F_{3,j}\times 44/28$$

where *R_A,j_* is the volatilization rate of ammonia and NOx from agricultural land for crop *j*; *R_L,j_*is the N leaching and runoff rate of farmland for crop *j*; *F*_2,*j*_ (kg N_2_O-N kg^−1^) is the indirect emission factor of N_2_O caused by nitrogen deposition for crop *j*; and *F*_3,*j*_ (kg N_2_O-N kg^−1^) is the indirect emission factor of N_2_O caused by leaching and runoff for crop *j*.

Furthermore, CH_4_ emissions from paddy fields in the carbon equivalent (*CE_CH4_*, kg CO_2_-eq · ha^−1^) were estimated using the equation:

(6) $$CE_{CH_{4}}=F_{CH_{4}}\times 28$$

where *F_CH4_* (kg CH_4_ ha^−1^) is the CH_4_ emission factor for paddy fields and 28 is the global warming potential of CH_4_ at the 100-year time horizon [26].

Therefore, total GHG emissions in the carbon equivalent for crop *j* (*CE_t,j_*, kg CO_2_-eq · ha^−1^) were calculated using the equation:

(7) $$CE_{t,j}=CE_{input,j}+CE_{N_{2}O,j}+CE_{CH_{4}}$$

Carbon footprint per unit area (*CF_A,j_*, kg CO_2_-eq · ha^−1^) and carbon footprint per unit yield (*CF_Y,j_*, kg CO_2_-eq · kg^−1^) for crop *j* in different provinces were calculated using the equations:

(8) $$CF_{A,j}=CE_{t,j}$$

(9) $$CF_{Y,j}=\frac{CE_{t,j}}{Y_{j}}$$

where *A_j_* is the sown area of crop *j* (ha) and *Y_j_* (kg ha^−1^) is the yield of crop *j*.

Furthermore, the total mean carbon footprint per unit area (m*CF_A_*, kg CO_2_-eq · ha^−1^) and CF per unit yield (m*CF_Y_*, kg CO_2_-eq · kg^−1^) for each province were calculated using the equations by considering all the crops together:

(10) $$mCF_{A}=\frac{\sum_{j=1}^{8}CE_{t,j}\times A_{j}}{\sum_{j=1}^{8}A_{j}}$$

(11) $$mCF_{Y}=\frac{\sum_{j=1}^{8}CE_{t,j}\times A_{j}}{\sum_{j=1}^{8}Y_{j}\times A_{j}}$$

### 2.5. Statistical Analysis

One-way ANOVA and the least significant difference test (LSD) at the level of 5% were used to compare the differences in the *CF_A_* and *CF_Y_* among the different crops. All statistical analyses were performed using R software [27].

## 3. Results

### 3.1. Temporal Dynamics of Carbon Footprint for Major Crops

**Figure 2:**
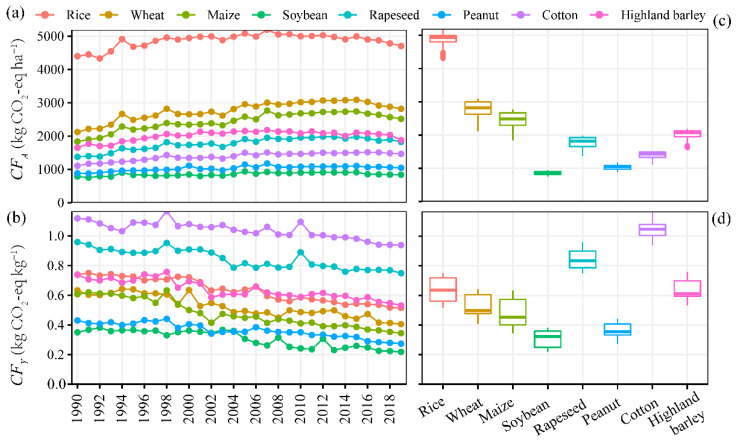
Temporal dynamics of the carbon footprint (**a**) per unit area and (**b**) per unit yield of major crops in China from 1990 to 2019, and the averaged carbon footprint (**c**) per unit area and (**d**) per unit yield of major crops in China.

The carbon footprint per unit area (*CF_A_*) showed significant differences for different crops regardless of interannual variation (Figure 2a), which were highest for rice (4333–5195 kg CO_2_-eq · ha^−1^), followed by wheat (2123–3091 kg CO_2_-eq · ha^−1^), maize (1838–2766 kg CO_2_-eq · ha^−1^), highland barley (1653–2182 kg CO_2_-eq · ha^−1^), rapeseed (1378–1984 kg CO_2_-eq · ha^−1^), cotton (1109–1516 kg CO_2_-eq · ha^−1^), peanut (878–1178 kg CO_2_-eq · ha^−1^), and soybean (753–940 kg CO_2_-eq · ha^−1^). Generally, the *CF_A_* for all major crops showed an increasing trend with time, which increased rapidly from 1990 to 1998, slowed down after 1999, and slowly decreased from 2016 to 2019.

In contrast, the carbon footprint per unit yield (*CF_Y_*) showed an overall decreasing trend with fluctuation in some years (Figure 2b), which was mainly due to the increase in crop yield with time. In 1998, however, some areas of China suffered from catastrophic floods, which resulted in an extremely low yield and then in turn, a higher *CF_Y_*. In 2003 and 2010, high temperature and drought led to a dramatic reduction in crop yield, and thus a higher *CF_Y_* for several crops. As influenced by crop yield, the rank of *CF_Y_* for major crops was not consistent with that of *CF_A_*, where cotton showed the highest *CF_Y_* (0.94–1.17 kg CO_2_-eq · kg^−1^), followed by rapeseed (0.75–0.96 kg CO_2_-eq · kg^−1^), highland barley (0.53–0.76 kg CO_2_-eq · kg^−1^), rice (0.51–0.75 kg CO_2_-eq · kg^−1^), wheat (0.40–0.64 kg CO_2_-eq · kg^−1^), maize (0.22–0.96 kg CO_2_-eq · kg^−1^), peanut (0.27–0.44 kg CO_2_-eq · kg^−1^), and soybean (0.22–0.38 kg CO_2_-eq · kg^−1^). The higher *CF_Y_* of cotton and rapeseed could mainly be attributed to their lower yield but higher fertilizer requirements.

### 3.2. Distribution of Carbon Footprint in Different Regions

**Figure 3:**
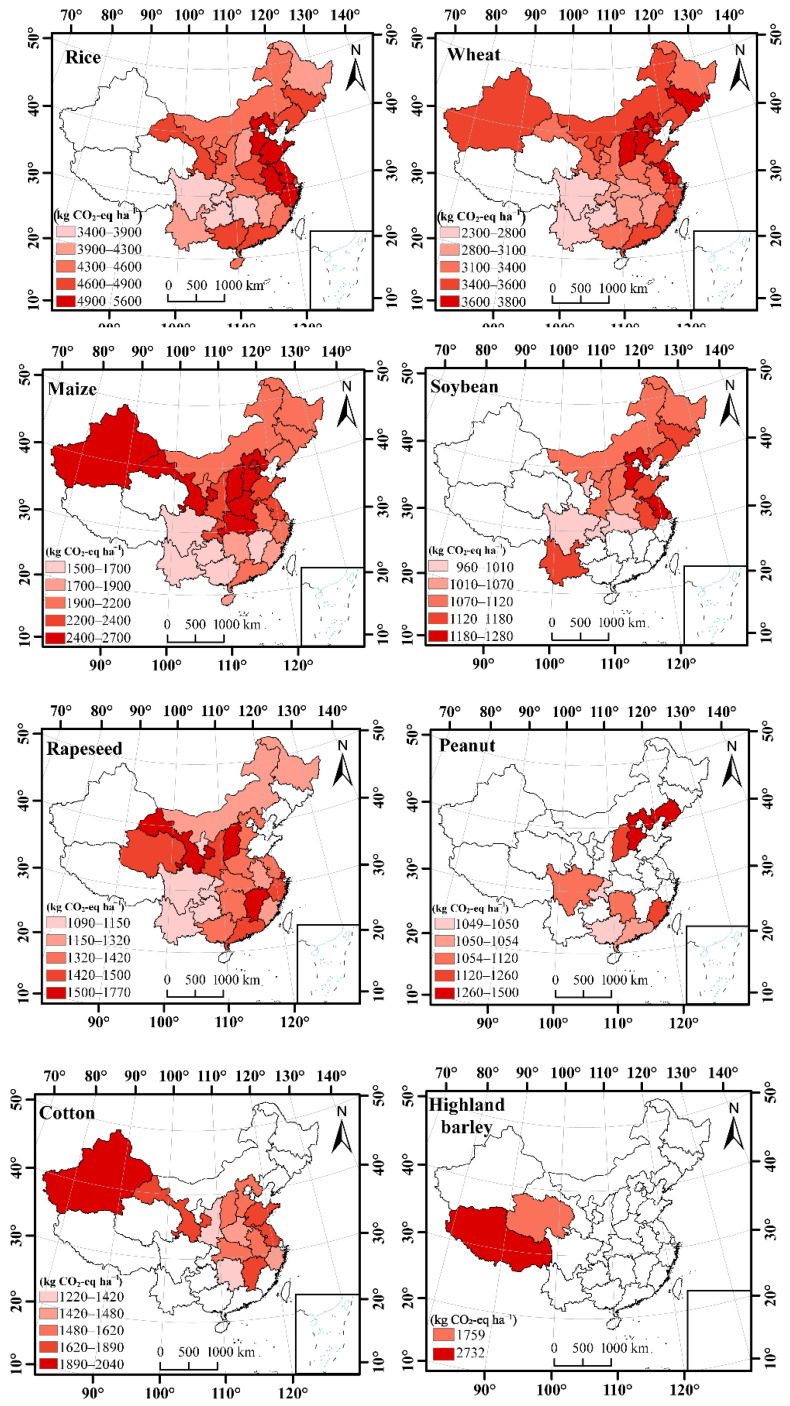
Spatial distributions of the annual mean carbon footprint per unit area (*CF_A_*) for different crops in China from 1990 to 2019.

**Figure 4:**
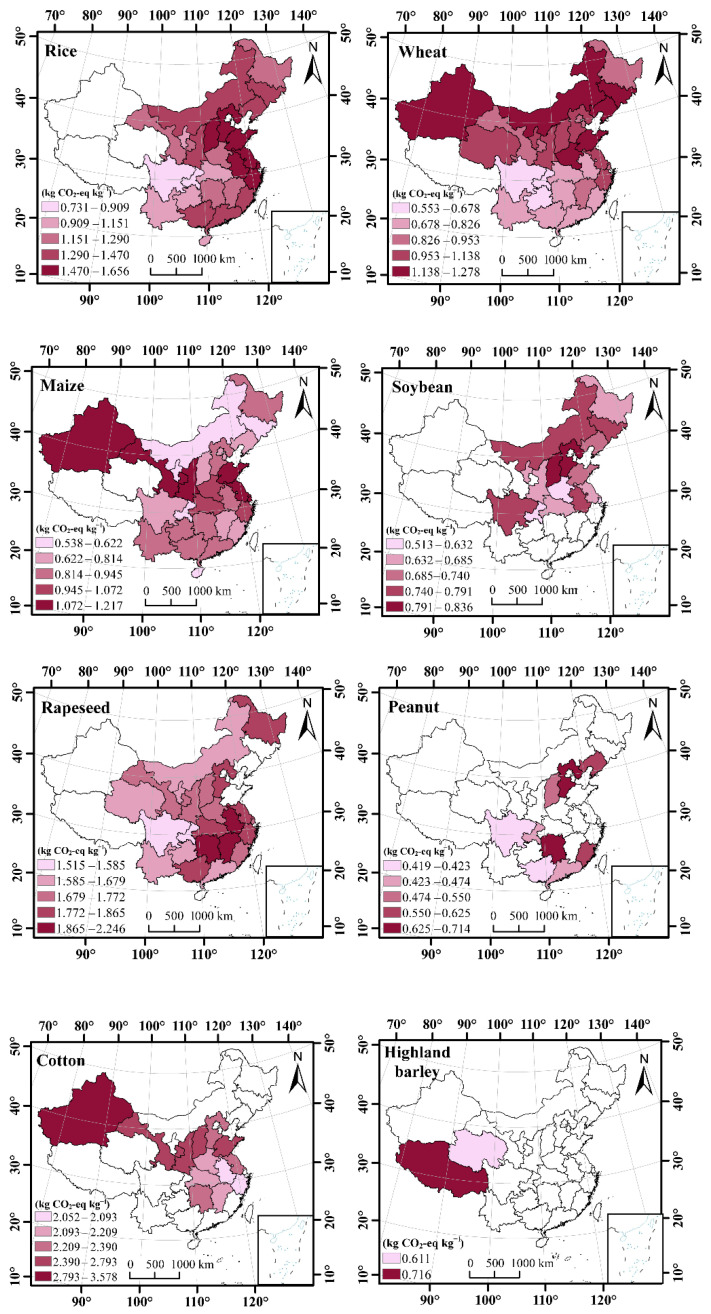
Spatial distributions of the carbon footprint per unit yield (*CF_Y_*) for different crops in China from 1990 to 2019.

The annual mean *CF_A_* and *CF_Y_* for major crops from 1990 to 2019 showed different spatial patterns due to the distributions of cropping area and yield across China (Figure 3 and Figure 4). For instance, the spatial distribution pattern of the annual mean *CF_A_* of wheat was higher in the northern provinces but lower in the southwest provinces, where the highest value of 3740 kg CO_2_-eq ha^−1^ was obtained in Shanxi province and the lowest value of 2361 kg CO_2_-eq ha^−1^ was obtained in Guizhou province (Figure 3). However, the annual mean *CF_A_* of soybean was higher in the eastern and southwest provinces but lower in the middle provinces of China, where the highest value of 1272 kg CO_2_-eq ha^−1^ was obtained in Jiangsu province and the lowest value of 966 kg CO_2_-eq ha^−1^ was obtained in Chongqing province.

**Figure 5:**
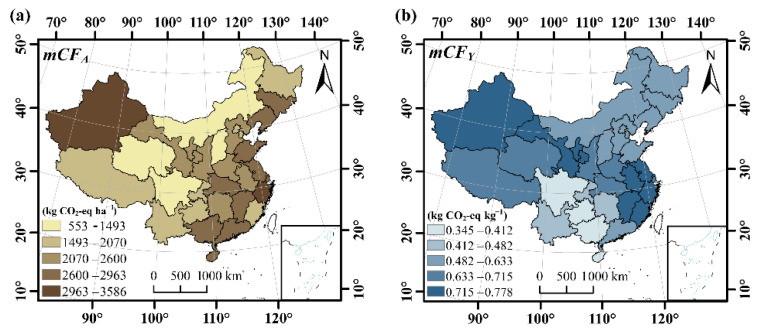
Spatial distributions of the annual total mean carbon footprint (**a**) per unit area and (**b**) per unit yield in China from 1990 to 2019.

In order to take all major crops together into consideration, the annual total mean carbon footprints (m*CF_A_* and m*CF_Y_*) for cropping lands in different provinces were calculated by averaging the values from 1990 to 2019 (Figure 5). The higher m*CF_A_* values of crop production were mainly obtained in Xinjiang province in the west, Zhejiang, Shandong, Jiangsu, and Zhejiang in the east, and Hubei, Guangdong, and Guangxi in the middle of China, where the m*CF_A_* was more than 2800 kg (CO_2_-eq) ha^−1^. Lower m*CF_A_* values of crop production were obtained in Qinghai, Guizhou, Sichuan, and Chongqing, where the m*CF_A_* was below 2000 kg (CO_2_-eq) ha^−1^. In general, the m*CF_A_* values were higher in the eastern and western provinces but lower in the middle provinces (Figure 5a). On the other hand, the m*CF_Y_* for major crops in China ranged from 0.34 to 0.78 kg (CO_2_-eq) kg^−1^ (Figure 5b). The m*CF_Y_* of 15 provinces, including Guangxi, Sichuan, Guizhou, and Yunnan, were lower than the national average of 0.61 kg (CO_2_-eq) kg^−1^, which were mainly obtained in the southwest and northeast regions of China. However, other provinces with a higher annual mean m*CF_Y_* were distributed in the northwest and eastern coastal regions of China.

### 3.3. Composition of Carbon Footprint for Different Crops

**Figure 6:**
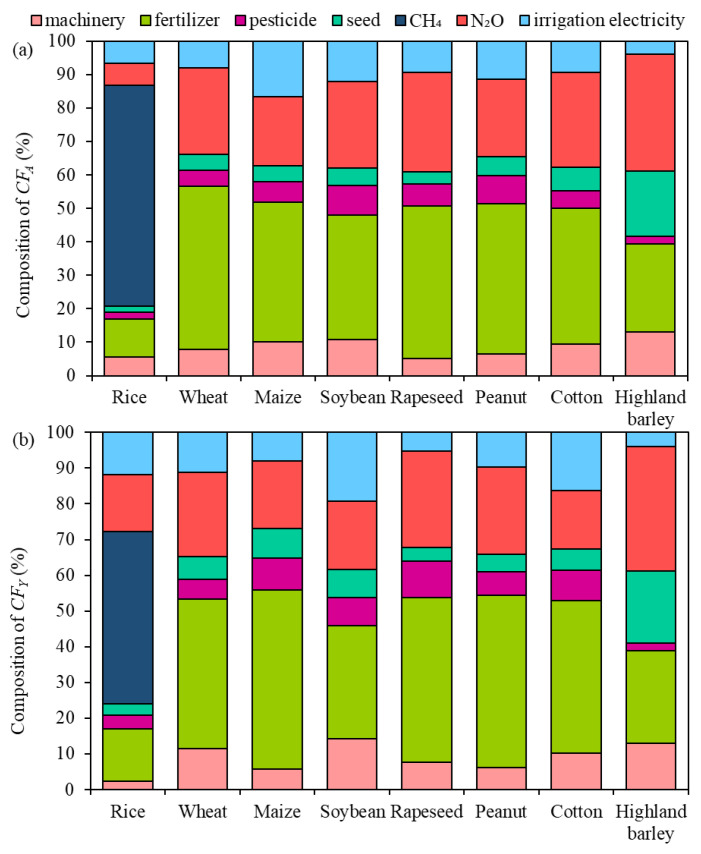
Compositions of the annual mean carbon footprints (**a**) per unit area and (**b**) per unit yield for different crops in China from 1990 to 2019.

According to the defined system boundary (Figure 1), the carbon footprint of crop production in this study consisted of the energy consumption by machinery operation, fertilizer production, pesticides, seeds, CH_4_ emission from paddy fields, N_2_O emission from N fertilizer application, and electricity consumption by irrigation. The relative contribution of these components to the *CF_A_* and *CF_Y_* for different provinces were calculated and their annual mean values from 1990 to 2019 are shown in Figures S1–S8 for different crops. Although the composition of the annual mean carbon footprint (both *CF_A_* and *CF_Y_*) of different crops showed slight differences among different provinces, the rank of these components was consistent for each crop (Figures S1–S8). Therefore, composition values of the *CF_A_* and *CF_Y_* were averaged across provinces to show differences among crops at a national scale. For rice production, CH_4_ emission was the major component of carbon footprint, accounting for 66% and 48% of *CF_A_* and *CF_Y_*, respectively (Figure 6). Fertilizer production and usage as well as N_2_O emission from N fertilizer application made up 11% and 7% of *CF_A_* for rice production, but 15% and 16% of *CF_Y_*, respectively.

For dryland crops, however, fertilizer production and usage were the largest composition of carbon footprint, making up 26–49% of *CF_A_* and 26–50% of *CF_Y_* for different crops, respectively (Figure 6 and Table S3). N_2_O emission from N fertilizer application was the second largest component of carbon footprint, representing 21–35% of *CF_A_* and 16–25% of *CF_Y_* for different crops, respectively. In contrast, the proportion of other components to the carbon footprint was comparatively small, accounting for 5–13% of *CF_A_* and 6–14% of *CF_Y_* for energy consumption by machinery operation, 2–9% of *CF_A_* and 2–10% of *CF_Y_* for pesticide application, 4–20% of *CF_A_* and 4–20% of *CF_Y_* for seeds, and 4–17% of *CF_A_* and 4–19% of *CF_Y_* for electricity consumption by irrigation, respectively.

## 4. Discussion

### 4.1. Dynamics of Carbon Footprint

**Figure 7:**
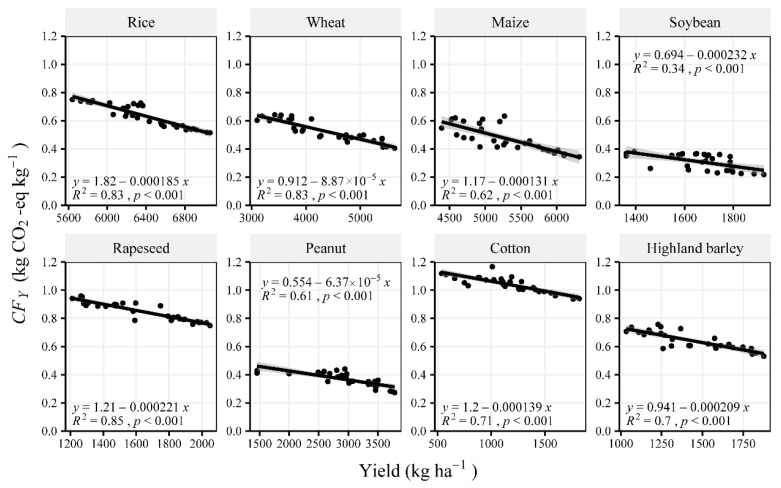
Correlations between the carbon footprint per unit yield (*CF_Y_*) and crop yield for the study period.

The fossil fuel CO_2_ emissions in China increased from 2.48 × 10^9^ t CO_2_-eq · in 1990 to 10.49 × 10^9^ t CO_2_-eq · in 2019 [28]. The carbon footprint of the eight major crop productions estimated in the present study corresponded to ~7% of the national total emissions, revealing the importance of GHG emissions from crop production in China. The *CF_A_* for all major crops showed an increasing trend with time before 2016 but slowly decreased afterward (Figure 2), which fluctuated as the cultivated area changed (Figure S9). This trend is in agreement with Wu*,* et al. [29], who found that national GHG emissions per unit area increased from 980 kg CO_2_-eq · ha^−1^ in 1998 to 1200 kg CO_2_-eq · ha^−1^ in 2016. In contrast, the *CF_Y_* decreased by 16–43% over the past 30 years for different crops (Figure 2b), which was significantly correlated with crop yield (Figure 7).

The *CF_A_* and/or *CF_Y_* for all major crops in this study (Figure 2) were markedly higher than those reported for other countries. For instance, the *CF_A_* of wheat was reported to be 350 kg CO_2_-eq · ha^−1^ in the UK [19], 110 kg CO_2_-eq · ha^−1^ in Denmark [30], and 90 kg CO_2_-eq · ha^−1^ in Australia [31], while the *CF_A_* of rapeseed was 220–350 kg CO_2_-eq · ha^−1^ in Canada [32] and the *CF_A_* of cotton was 350–370 kg CO_2_-eq · ha^−1^ in Australia [33]. On the other hand, the *CF_Y_* of rice was reported to be 0.07–0.10 kg CO_2_-eq · kg^−1^ in the USA [34], while the *CF_Y_* of wheat was 0.07–0.14 kg CO_2_-eq · kg^−1^ in Canada [35]. The *CF_Y_* of maize was 0.06 kg CO_2_-eq · kg^−1^ in the USA [36] and the *CF_Y_* of cotton was 0.43–0.48 kg CO_2_-eq · kg^−1^ in Australia [33]. Higher *CF_A_* and *CF_Y_* values in China provide a greater potential for mitigating GHG emissions in Chinese cropland.

The *CF_A_* and *CF_Y_* for all dryland crops in the present study were dramatically dependent on chemical fertilizer, including chemical fertilizer production, transportation, and N_2_O emissions from chemical fertilizer use, which together contributed to 61–74% of the *CF_A_* and 51–73% of the *CF_Y_*, respectively (Figure 6). These values were comparable with those reported by Cheng, et al. [17] (57%) from 1993 to 2007, and Liu*,* et al. [37] (50.7%) from 2000 to 2015 in China, but lower than the mean proportion of 75% for UK croplands in 2007 [19]. Therefore, the GHG emissions from fertilizer that were produced and then used on cropland contributed the most to the GHG emissions of dryland crop production [38]. During the study period, the *CF_A_* reached the highest value in 2015 and decreased afterward (Figure 2a), resulting from the decrease in the chemical fertilizer application amount by 10% from 2015 to 2019 (Figure S10) by the implementation of the “action plan for zero growth in fertilizer use by 2020” at a national scale since 2015. Our results indicated that a cut in chemical fertilizer use and adoption of science-based fertilizer applications in crop production in China will offer a great option to reduce the national total GHG emissions. Furthermore, controlling N leaching, runoff, and volatilization losses after application by using deep placement and/or other techniques could also mitigate GHG emissions [39]. In addition, the use of organic fertilizer and other amendments, such as N-saving and slow-release biochar-based fertilizers could reduce GHG emissions and increase the crop yield, which further offer measures to cut down the carbon footprint from chemical fertilizer production and usage in China [37,40,41].

### 4.2. Carbon Footprint for Different Crops and GHG Mitigation Suggestions

Our results show that the *CF_A_* values of rice, wheat, and maize are generally higher than those of other crops (soybean, rapeseed, peanut, cotton, and highland barley; Figure 2a). Lin*,* et al. [42] also found that rice, maize, and vegetables were the top three contributors to the grain carbon footprint in 2009. Compared with other dryland crops, CH_4_ emission from paddy fields is the biggest contributor to the *CF_A_* of rice (Figure 6). Rice is the crop with the highest fertilizer application rate, of ~300 kg N ha^−1^ in China [43], among the eight studied crops. However, it has been reported that N fertilizer application above 225 kg N ha^−1^ had little, or even a negative, impact on the rice yield and would increase GHG emissions [44]. Zhang*,* et al. [45] found that a 10% reduction in chemical N fertilizer in paddy fields relative to the current farmer’s application rate (300 kg N ha^−1^) could realize high yields and N use efficiency as well as low environmental impacts. In addition, rice production consumes more electricity per unit area than other crops for irrigation, which is also an important factor leading to a higher carbon footprint for rice. Solar energy-assisted electricity is suggested as a renewable energy source for irrigation [46].

Rice, wheat, and maize have the three largest cultivated areas in China (Figure S9), which further strongly affect the spatial distribution of the total carbon footprint (Figure 5). A reduction in the cultivated areas of these crops would remarkably reduce their carbon footprint [29], but would compromise national food supplies and food security in China. In 2018, the Ministry of Agriculture of China issued “Key Points of Planting in 2018”, which plan to reduce the rice area by 2–3%, stabilize or reduce the wheat area by 1%, and reduce the maize area by 9–10%, to improve their production efficiencies. However, these plans are mainly being implemented in the regions with low productivity, high resource consumption, and insect damage.

In contrast, soybean, rapeseed, peanut, cotton, and highland barley contributed a relatively much lower carbon footprint during the study period, mainly due to the significantly smaller cultivated areas of these five crops (Figure S9). On the other hand, their fertilizer application rates were also much smaller, except for cotton. For instance, the fertilizer application rate of oil crops is only about 200 kg · ha^−1^. Zhang*,* et al. [47] found that the rapeseed production mode could be more efficient by adopting direct sowing, machine sowing, and harvesting. However, soybean is more energetically efficient than rapeseed due to its lower N fertilizer requirement [48]. China is the second largest consumer of oil crops in the world but is reliant on imports. In 2021, China produced 16.4 million tons of soybeans, which met the needs of edible consumption, but also imported 96.5 million tons, mainly used to make edible oils and animal fodder. Therefore, increasing the yield of oil crops, especially soybean, without increasing either fertilizer use or cultivated areas may be an important priority in their production to reduce the dependency on imports.

Although both the cultivated area (Figure S9) and yield of cotton were the smallest among all the crops, cotton had the highest *CF_Y_* (Figure 2b) due to its high fertilizer application rate. Ma*,* et al. [49] suggested that plastic mulch and drip irrigation in cotton production could significantly decrease N_2_O emissions as well as improve water use efficiency. Powell*,* et al. [50] found that rotating cotton with pulse crops, instead of wheat, could reduce GHG emissions by 8%. In China, 76% of cotton was cultivated in Xinjiang province in 2019, where the soil conditions such as low soil organic matter, light soil texture, and high soil pH, as well as climate factors of low precipitation and high evapotranspiration, restricted soil N_2_O emissions. Therefore, decreasing fertilizer rates or replacing them with polymer-coated urea while improving cotton yields with more efficient production techniques should be considered by future cotton producers.

However, different strategies should be considered for different provinces. For instance, reducing chemical N fertilizer use and controlling the pathways of N loss were important GHG mitigation strategies across China [39,51]. Reduction in CH_4_ emissions from paddy fields should be considered in the south, while conservation tillage measures should be implemented to mitigate future increases in GHG emissions in the north. Therefore, proper management could enhance carbon sequestration, reduce GHG emissions, as well as increase crop yields simultaneously, and subsequently decrease the carbon footprint of crop production [37,52].

## 5. Conclusions

In this study, the spatiotemporal dynamics of the carbon footprint of major crops in China from 1990 to 2019 were investigated to quantify the mitigation potential of management strategies in Chinese agriculture. Our results showed that the carbon footprint of the eight major crop productions estimated in the present study corresponded to ~7% of the national total emissions, revealing the importance of GHG emissions from crop production in China. The *CF_A_* and/or *CF_Y_* for all major crops in this study were markedly higher than those reported for other countries, which provided greater potential for mitigating GHG emissions in Chinese cropland. CH_4_ emission from paddy fields is the biggest contributor to the *CF_A_* of rice, followed by fertilizer production and usage, while the *CF_A_* and *CF_Y_* for all dryland crops were dramatically dependent on chemical fertilizer. Therefore, cutting chemical fertilizer use and adopting science-based fertilizer applications, controlling N leaching, runoff, and volatilization losses after application by using deep placement and/or other techniques, as well as using organic fertilizer and other amendments, will offer a great option to cut down the carbon footprint of crop production in China.

## Data Availability

The data that support the findings of this study are available upon request.

## Supplementary Materials

The following supporting information can be downloaded at: https://www.mdpi.com/article/10.3390/ijerph192113896/s1, Figure S1: Composition of the annual mean carbon footprint per unit area (*CF_A_*) and per unit yield (*CF_Y_*) of rice production for different provinces from 1990 to 2019; Figure S2: Composition of the annual mean carbon footprint per unit area (*CF_A_*) and per unit yield (*CF_Y_*) of wheat production for different provinces from 1990 to 2019; Figure S3: Composition of the annual mean carbon footprint per unit area (*CF_A_*) and per unit yield (*CF_Y_*) of maize production for different provinces from 1990 to 2019; Figure S4: Composition of the annual mean carbon footprint per unit area (*CF_A_*) and per unit yield (*CF_Y_*) of soybean production for different provinces from 1990 to 2019; Figure S5: Composition of the annual mean carbon footprint per unit area (*CF_A_*) and per unit yield (*CF_Y_*) of rapeseed production for different provinces from 1990 to 2019; Figure S6: Composition of the annual mean carbon footprint per unit area (*CF_A_*) and per unit yield (*CF_Y_*) of peanut production for different provinces from 1990 to 2019; Figure S7: Composition of the annual mean carbon footprint per unit area (*CF_A_*) and per unit yield (*CF_Y_*) of cotton production for different provinces from 1990 to 2019; Figure S8: Composition of the annual mean carbon footprint per unit area (CF_A_) and per unit yield (CF_Y_) of highland barley production for different provinces from 1990 to 2019; Figure S9: Changes in the cultivated area of major crops in China from 1990 to 2019; Figure S10: Changes in the chemical fertilizer application amount in China from 1990 to 2019; Table S1: Annual averaged sown area of major crops for different provinces in China during 1990–2019; Table S2: Greenhouse gas emission factors for agriculture inputs; Table S3: Composition of the annual mean *CF_A_* and *CF_Y_* for different crops in China from 1990 to 2019.
