# Supplementary material for: Spatiotemporal Dynamics of Carbon Footprint of Main Crop Production in China
Source: Int J Environ Res Public Health. 2022 Oct 26;19(21):13896. doi: 10.3390/ijerph192113896 (PMC9658409; doi:10.3390/ijerph192113896)
Supplement: Supplementary file 1 [file ijerph-19-13896-s001.zip › ijerph-1970611-supplementary.pdf]

Supplementary Materials for Spatiotemporal Dynamics of Carbon Footprint of Main Crops Production in China

Table S1. Annual averaged sown area of major crops for different provinces in China during 1990-2019.

Province	Annual Average Sown Area of Major Crops in China from 1990 to 2019 (Unit: 10^3 ha)								Total Sown Area	Proportion (%) *
	Rice	Wheat	Maize	Soybean	Rapeseed	Peanut	Cotton	Highland Barley		
Beijing	/	89	140	/	/	/	/	/	264	87
Tianjin	/	118	165	/	/	/	/	/	385	73
Hebei	106	2452	2724	372	/	386	551	/	6966	95
Shanxi	4	792	1177	353	/	16	77	/	2708	89
Inner Mongoria	96	726	2029	939	198	/	/	/	4509	89
Liaoning	553	67	1858	235	/	198	/	/	3034	96
Jilin	611	34	2897	478	/	/	/	/	4211	95
Heilongjiang	2054	584	3460	189	22	/	/	/	9596	66
Shanghai	148	51	5	7	38	/	/	/	256	98
Jiangsu	2241	2106	439	323	445	/	336	/	6150	96
Zhejiang	1347	141	49	/	202	/	35	/	2065	86
Anhui	2221	2250	698	781	750	/	336	/	7588	93
Fujian	1059	33	36	/	14	100	/	/	1639	76
Jiangxi	3148	35	25	/	534	/	88	/	4306	89
Shandong	132	3757	2850	319	/	/	769	/	9074	86
Henan	546	5104	2650	574	289	/	669	/	11232	88
Hubei	2199	1042	490	252	935	/	410	/	5865	91
Hunan	3989	92	259	/	852	119	155	/	6017	91
Guangdong	2258	16	133	/	9	340	/	/	3263	85
Guangxi	2217	12	559	/	47	209	/	/	3562	85
Hainan	338	/	20	/	/	/	/	/	519	69
Chongqing	547	181	361	165	148	50	/	/	2177	67
Sichuan	2099	1515	1455	464	833	234	/	/	8112	81
Guizhou	710	392	707	/	427	/	/	/	3326	67
Yunnan	982	526	1255	/	171	/	/	/	3969	74
Tibet	/	/	/	/	/	/	/	124	201	62
Shaanxi	135.9	1317	1088	309	173	/	62	/	3476	89
Gansu	5.6.0	1060	619	/	143	/	37	/	2583	72
Qinghai	/	143	/	/	146	/	/	76	494	74
Ningxia	72	238	186	60	1	/	/	/	684	82
Xinjiang	/	971	612	/	/	/	1332	/	3159	92

* The proportion of the total sown area of main crops in the total sown area of all provinces, with Heilongjiang, Hainan, Chongqing, Guizhou and Tibet accounting for less than 70%, mainly due to the large annual sown area of vegetables and sugar crops in Heilongjiang (220.7 thousand ha), fruits and vegetables in Hainan (389.1 thousand ha), vegetables and fruits in Chongqing (837.8, thousand ha), tea gardens and vegetables in Guizhou (799.7 thousand ha), and vegetables in Tibet (21.3 thousand ha).

Table S2. Greenhouse gas emission factors for agriculture inputs.

Agricultural Capital Investment	Data Sources	Emission Factors（Unit：kg kg^{-1}）		
		CO_2	CH_4	N_2O
Rice seed	Ecoinvent 3.8	1.70E-2	1.07E-2	3.05E-4
Wheat seed	Ecoinvent 3.8	9.88E-3	1.08E-3	1.72E-5
Maize seed	Ecoinvent 3.8	4.42E-2	1.06E-3	4.75E-4
Soybean seed	Ecoinvent 3.8	4.58E+0	1.06E-3	7.14E-4
Rapeseed seed	Ecoinvent 3.8	1.43E-2	1.52E-3	1.16E-3
Peanut seed	Ecoinvent 3.8	3.62E-2	1.02E-3	7.60E-4
Cotton seed	Ecoinvent 3.8	3.35E-1	6.08E-3	1.42E-3
Highland barley	Ecoinvent 3.8	1.70E-02	1.07E-02	3.05E-04
Nitrogen fertilizer (Urea)	Ecoinvent 3.8	3.80E-2	6.83E-3	1.16E-3
phosphate fertilizer (P_2O_5)	Ecoinvent 3.8	1.10E-1	1.47E-2	1.13E-4
potassic fertilizer (K_2O)	Ecoinvent 3.8	7.53E-2	9.40E-4	3.05E-4
herbicide	Ecoinvent 3.8	3.06E-1	2.50E-2	1.44E-4
fungicide	Ecoinvent 3.6	1.68E-1	1.81E-2	1.02E-4
pesticide	Ecoinvent 3.6	4.93E-1	7.19E-5	2.99E-4
Diesel	Ecoinvent 3.8	6.79E+0	7.08E-4	2.35E-4

Petrol	Ecoinvent 3.8	1.39E+0	3.94E-2	3.55E-5
Raw coal	Yao[1]	2.03E+1	3.94E-2	6.15E-5
Natural gas	Ecoinvent 3.7	2.81E-6	1.77E-4	3.62E-5
Fuel oil	Ecoinvent 3.7	9.27E-2	9.06E-4	2.43E-4
Mechanical production	Ecoinvent 3.7	1.36E-1	5.63E-6	5.23E-6
mechanical transportation	Ecoinvent 3.7	2.76E-1	4.21E-4	2.74E-3
Steel	Ecoinvent 3.7	6.27E-2	5.45E-3	4.06E-5
Rubber	Ecoinvent 3.7	3.02E+0	4.32E-3	1.63E-5
Gasoline truck	Ecoinvent 3.7	1.36E-1	5.63E-6	5.23E-6
Diesel truck	Ecoinvent 3.7	1.40E-4	3.40E-9	5.00E-9
rail transport	Ecoinvent 3.7	2.09E-2	8.66E-7	6.66E-7
shipping	Ecoinvent 3.7	5.05E-3	7.94E-8	2.57E-7

Table S3. Composition of annual mean CF_A and CF_Y for different crops in China from 1990 to 2019.

	Crop	Machinery	Fertilizer	Pesticides	Seed	CH_4	N_2O Emissions	Electricity
CF_A	rice	5.5 d	11.4 g	2.2 d	1.7 e	66.0	6.6 f	6.6 e
	wheat	7.8 c	48.7 a	4.8 c	4.8 c	0	25.8 c	8.1 de
	maize	10.2 ab	41.7 cd	6.2 b	4.7 c	0	20.6 e	16.6 a
	soybean	10.7 ab	37.2 e	9 a	5.1 c	0	25.8 cd	12.2 b
	rapeseed	5.1 d	45.7 b	6.6 b	3.6 d	0	29.7 b	9.3 cd
	peanut	6.4 cd	45 bc	8.4 a	5.6 c	0	23.2 de	11.3 bc
	cotton	9.4 b	40.6 d	5.1 c	7 b	0	28.5 b	9.3 cd
	highland barley	13.1 a	26.4 f	2.1 d	19.6 a	0	35 a	3.8 e
CF_Y	rice	2.4 e	14.5 f	3.7 e	3.2 f	48.3	15.9 e	12 c
	wheat	11.5 b	41.8 d	5.5 d	6.5 c	0	23.7 c	11.1 cd
	maize	5.7 d	50.2 a	8.9 ab	8.4 b	0	18.8 d	8 e
	soybean	14.2 a	31.6 e	8 bc	7.9 b	0	19 d	19.4 a
	rapeseed	7.6 c	46.2 bc	10.3 a	3.7 ef	0	26.9 b	5.3 f
	peanut	6.1 cd	48.4 ab	6.5 cd	4.9 de	0	24.5 bc	9.6 d
	cotton	10.1 b	42.8 cd	8.4 abc	6 cd	0	16.4 de	16.3 b
	highland barley	12.9 ab	26 e	2.1 e	20.2 a	0	34.9 a	3.9 f

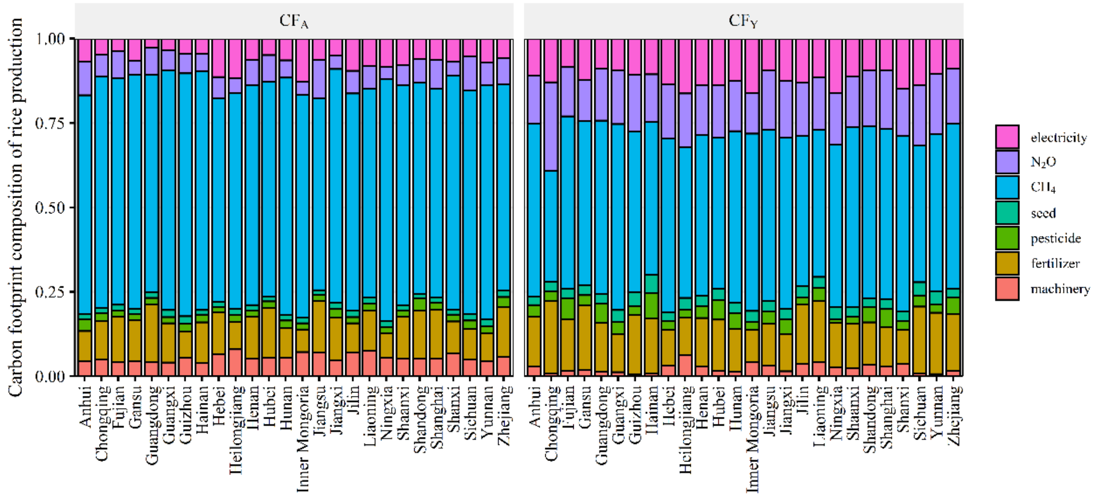

Figure S1. Composition of annual mean carbon footprint per unit area (CF_A) and per unit yield (CF_Y) of rice production for different provinces from 1990 to 2019.

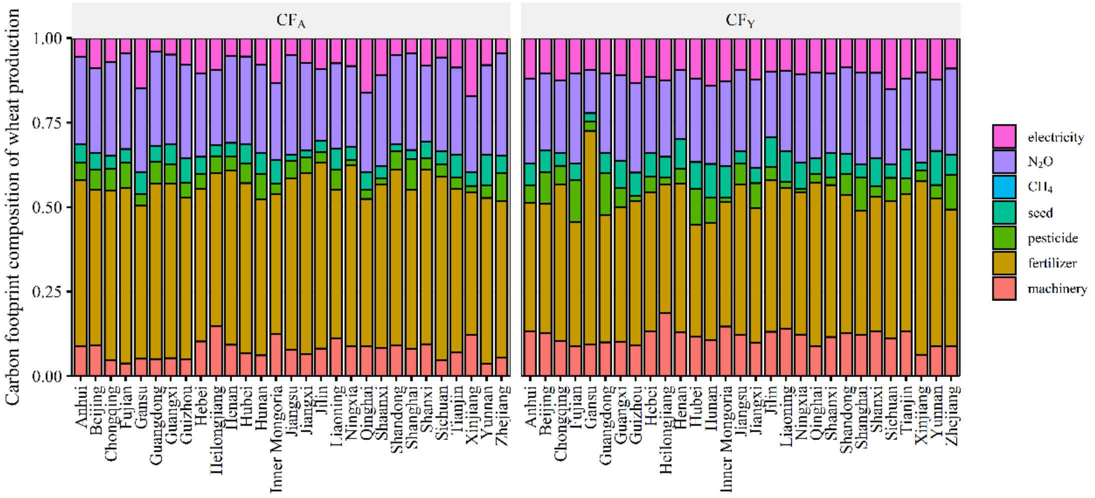

Figure S2. Composition of annual mean carbon footprint per unit area (*CFA*) and per unit yield (*CFY*) of wheat production for different provinces from 1990 to 2019.

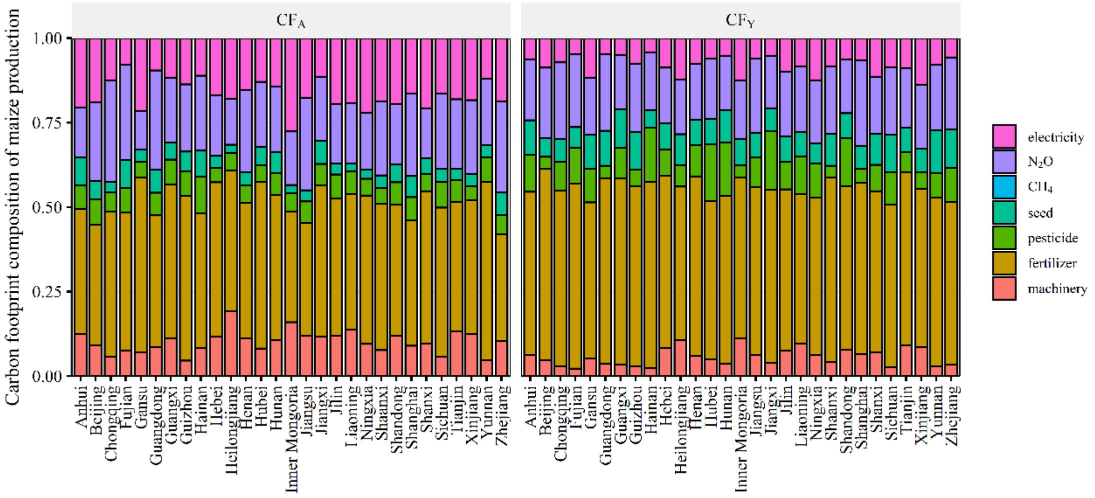

Figure S3. Composition of annual mean carbon footprint per unit area (*CFA*) and per unit yield (*CFY*) of maize production for different provinces from 1990 to 2019.

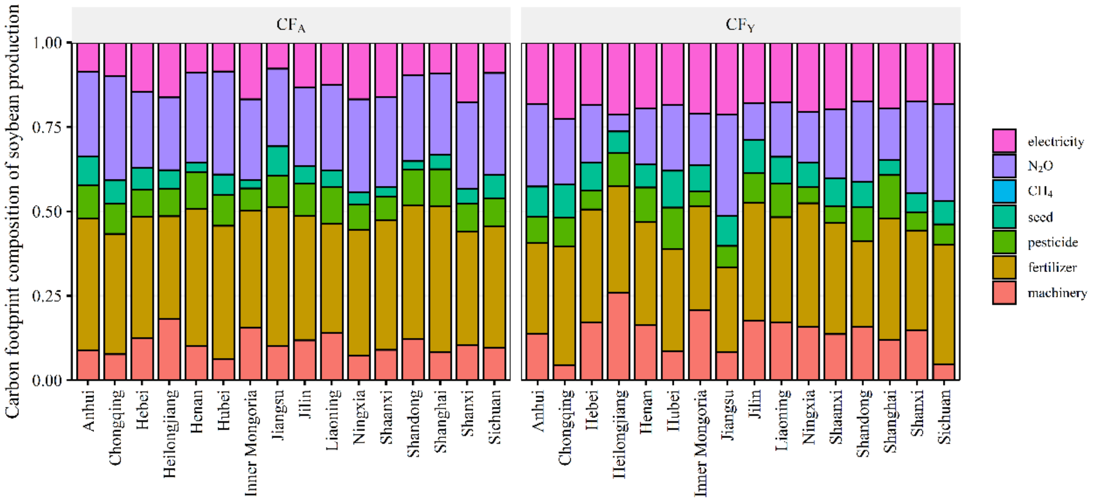

Figure S4. Composition of annual mean carbon footprint per unit area (*CFA*) and per unit yield (*CFY*) of soybean production for different provinces from 1990 to 2019.

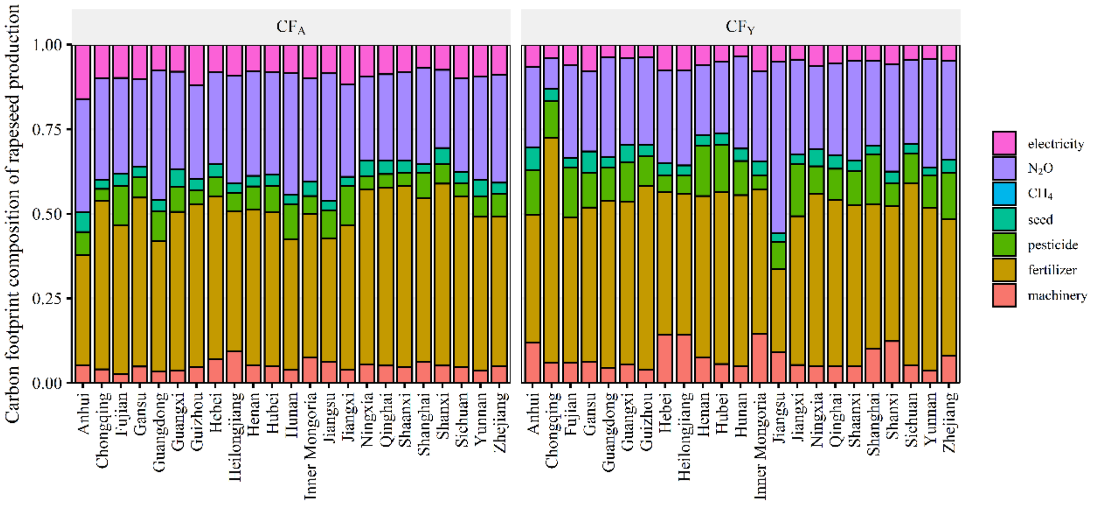

Figure S5. Composition of annual mean carbon footprint per unit area (CF_A) and per unit yield (CF_Y) of rapeseed production for different provinces from 1990 to 2019.

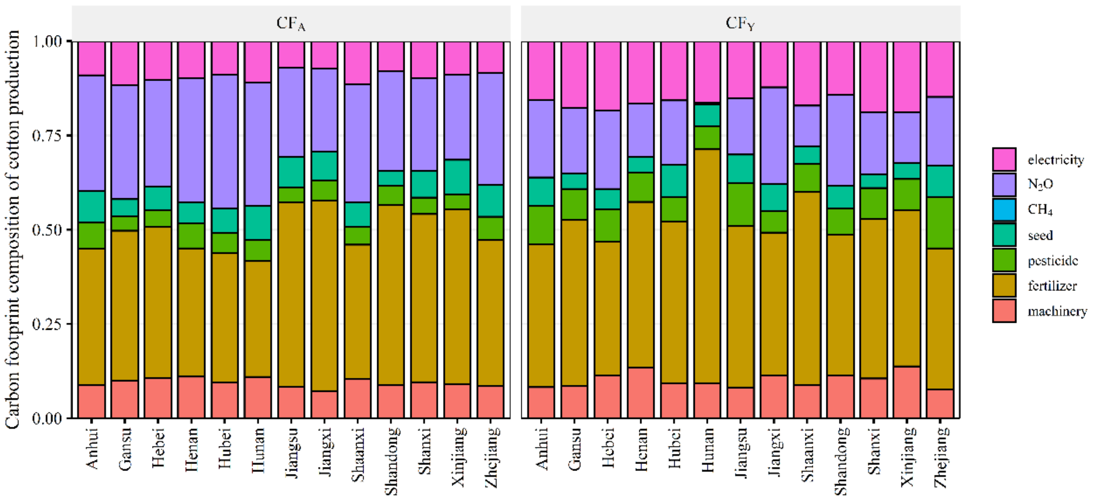

Figure S6. Composition of annual mean carbon footprint per unit area (CF_A) and per unit yield (CF_Y) of peanut production for different provinces from 1990 to 2019.

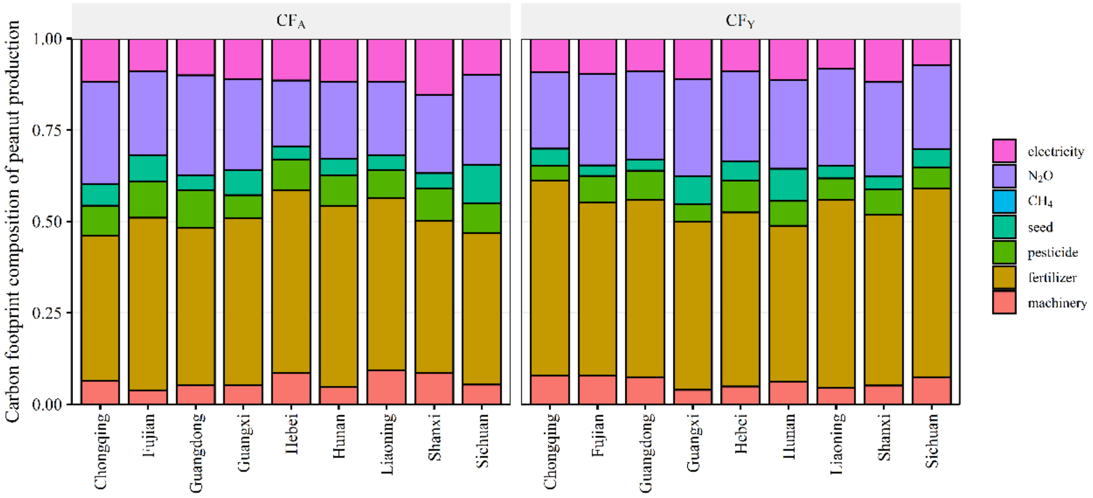

Figure S7. Composition of annual mean carbon footprint per unit area (CF_A) and per unit yield (CF_Y) of cotton production for different provinces from 1990 to 2019.

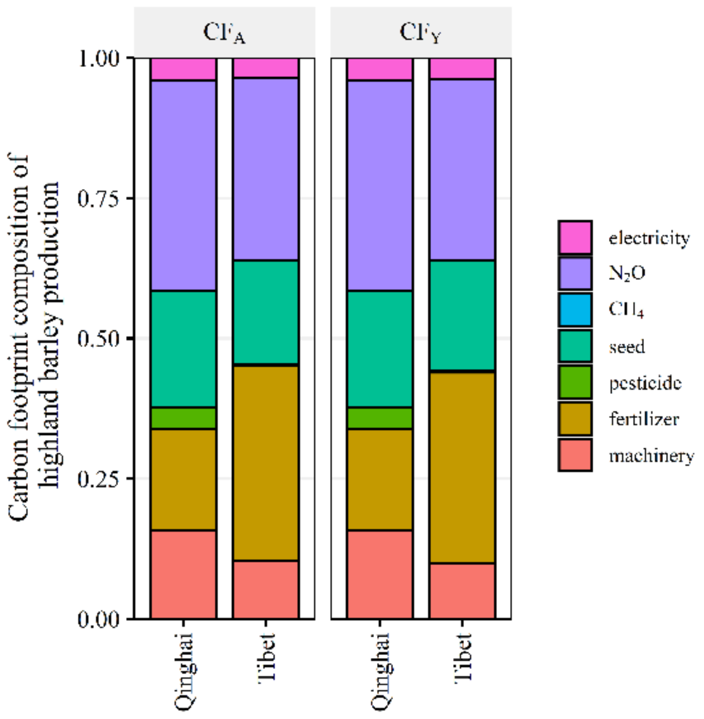

Figure S8. Composition of annual mean carbon footprint per unit area (CF_A) and per unit yield (CF_Y) of highland barley production for different provinces from 1990 to 2019.

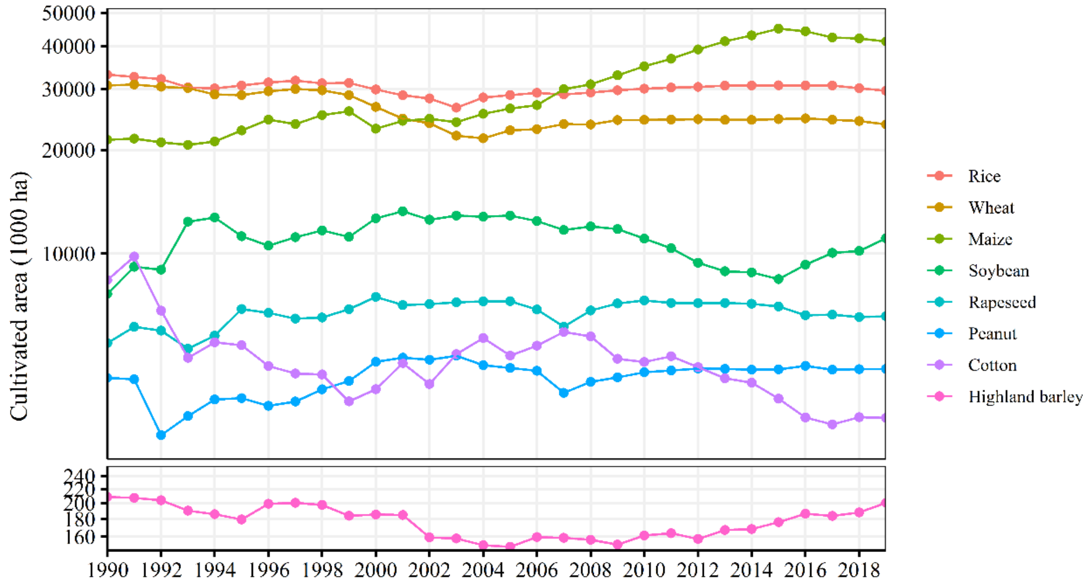

Figure S9. Change in cultivated area of major crops in China from 1990 to 2019.

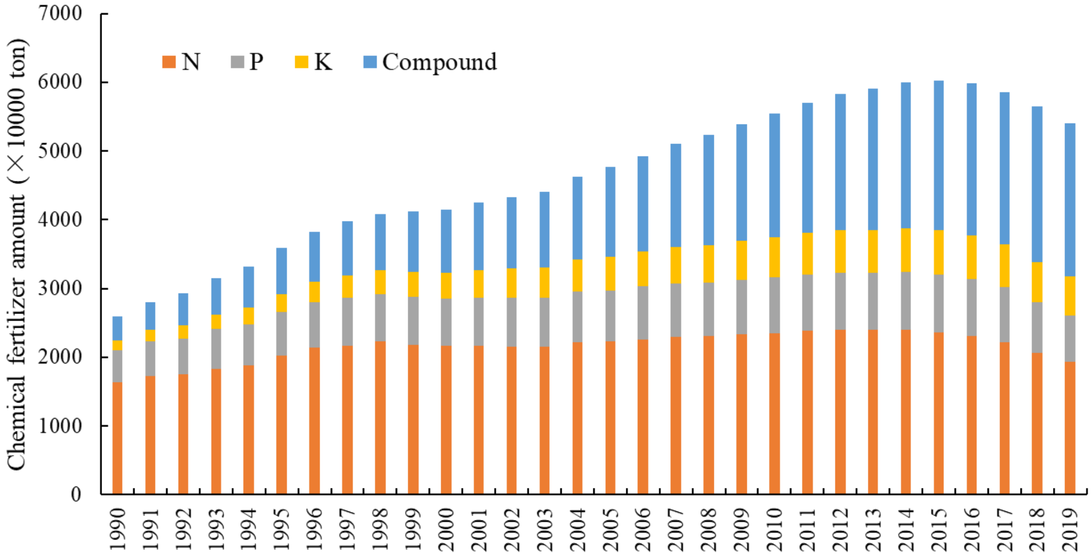

Figure S10. Change in chemical fertilizer application amount in China from 1990 to 2019.

References:

1. Yao, Y.; Chang, Y.; Huang, R.Z.; Zhang, L.X. Environmental implications of the methanol economy in China: well-to-wheel comparison of energy and environmental emissions for different methanol fuel production pathways. *Journal of Cleaner Production* **2018**, *172*, 1381-1390.
